# Stable Production of the Antimalarial Drug Artemisinin in the Moss *Physcomitrella patens*

**DOI:** 10.3389/fbioe.2017.00047

**Published:** 2017-08-15

**Authors:** Nur Kusaira Binti Khairul Ikram, Arman Beyraghdar Kashkooli, Anantha Vithakshana Peramuna, Alexander R. van der Krol, Harro Bouwmeester, Henrik Toft Simonsen

**Affiliations:** ^1^Faculty of Science, Institute of Biological Sciences, University of Malaya, Kuala Lumpur, Malaysia; ^2^Department of Plant and Environmental Sciences, University of Copenhagen, Frederiksberg, Denmark; ^3^Department of Biotechnology and Biomedicine, Technical University of Denmark, Kongens Lyngby, Denmark; ^4^Laboratory of Plant Physiology, Wageningen University, Wageningen, Netherlands; ^5^Plant Hormone Biology Lab, Swammerdam Institute for Life Sciences, University of Amsterdam, Amsterdam, Netherlands

**Keywords:** *Physcomitrella patens*, malaria, artemisinin, *in vivo* assembly, bioengineering

## Abstract

Malaria is a real and constant danger to nearly half of the world’s population of 7.4 billion people. In 2015, 212 million cases were reported along with 429,000 estimated deaths. The World Health Organization recommends artemisinin-based combinatorial therapies, and the artemisinin for this purpose is mainly isolated from the plant *Artemisia annua*. However, the plant supply of artemisinin is irregular, leading to fluctuation in prices. Here, we report the development of a simple, sustainable, and scalable production platform of artemisinin. The five genes involved in artemisinin biosynthesis were engineered into the moss *Physcomitrella patens via* direct *in vivo* assembly of multiple DNA fragments. *In vivo* biosynthesis of artemisinin was obtained without further modifications. A high initial production of 0.21 mg/g dry weight artemisinin was observed after only 3 days of cultivation. Our study shows that *P. patens* can be a sustainable and efficient production platform of artemisinin that without further modifications allow for industrial-scale production. A stable supply of artemisinin will lower the price of artemisinin-based treatments, hence become more affordable to the lower income communities most affected by malaria; an important step toward containment of this deadly disease threatening millions every year.

## Introduction

*Physcomitrella patens* is a non-vascular plant that has been well established as a model organism to be used in basic research and in applied biotechnology (Simonsen et al., 2009; Buttner-Mainik et al., 2011; Ikram et al., 2015; Reski et al., 2015). The genome is fully sequenced and the haploid life cycle and efficient homologous recombination makes *P. patens* an attractive industrial production system compared to other plant hosts (Schaefer and Zrÿd, 1997; Reski, 1998). Additionally, a novel transformation technology involving *in vivo* assembly of multiple DNA fragments in *P. patens* has been established, further increasing the potential as a photosynthetic chassis for synthetic biology (King et al., 2016). Currently, several recombinant pharmaceutical proteins and molecules of commercial value are being produced in this system (Anterola et al., 2009; Zhan et al., 2014; Pan et al., 2015; Reski et al., 2015).

Artemisinin originates from the plant *Artemisinia annua* and is the first-choice treatment for malaria (Novotny et al., 1966). Chemically, artemisinin is a sesquiterpene lactone bearing a unique endoperoxide structure and its complex structure makes it difficult and not economically feasible to be chemically synthesized (Pandey and Pandey-Rai, 2016). Several efforts have been made to obtain artemisinin from a stable source, such as yeast (Ro et al., 2006), where a production of artemisinic acid followed by a three-step chemical synthesis to artemisinin was established (Paddon and Keasling, 2014). It was previously shown that artemisinin could be mass-produced by cultivation of *A. annua* or semi-synthetically in microorganisms producing artemisinic acid (Paddon and Keasling, 2014). Besides these efforts, extensive bioengineering of modified *Nicotiana* plants (Malhotra et al., 2016; Wang et al., 2016) has provided limited production of artemisinin. However, these have not yet been used for large-scale production. Currently, there are no reports on the stable heterologous biosynthesis of artemisinin in photosynthetic organisms that can be grown in bioreactors.

**Figure 1:**
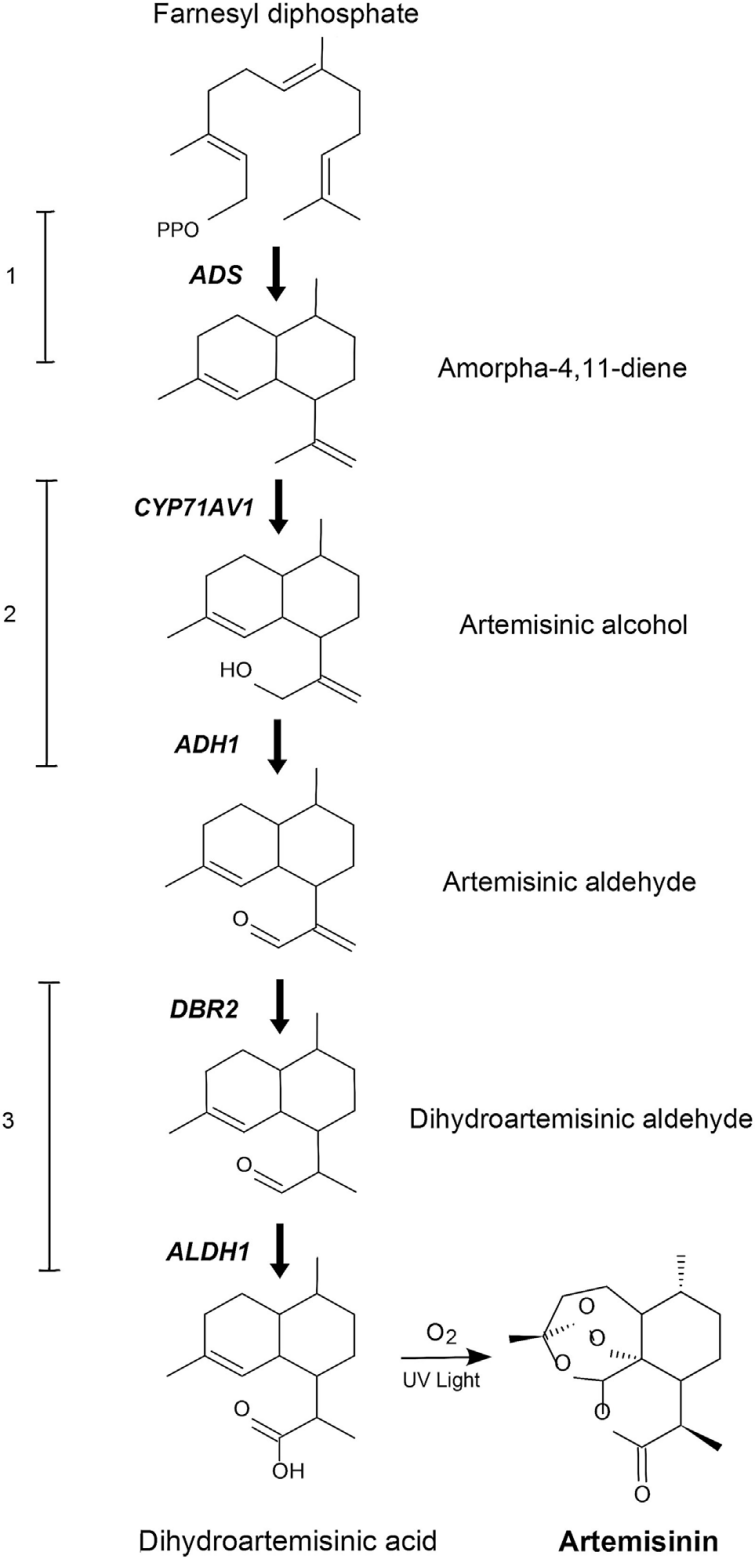
Schematic representation of the engineered artemisinin biosynthetic pathway in *Physcomitrella patens*. The five genes that encode the dihydroartemisinic acid biosynthetic pathway are *ADS*, amorphadiene synthase; *CYP71AV1*, amorphadiene oxidase; *ADH1*, alcohol dehydrogenase 1; *DBR2*, artemisinic aldehyde double-bond reductase; *ALDH1*, aldehyde dehydrogenase 1. The numbers 1–3 indicates transformation sets.

In order to produce artemisinin in *P. patens*, we introduced the five genes responsible for the biosynthesis of dihydroartemisinic acid (Figure 1), where amorpha-4,11-diene synthase, *ADS* (Komatsu et al., 2010) catalyzes the first step. This was followed by the second step, the cytochrome P450, *CYP71AV1* (Ro et al., 2006) which we linked with the third step, the alcohol dehydrogenase 1 (*ADH1*) (Paddon et al., 2013) by the hybrid LP4/2A peptide linker (François et al., 2004). Finally, we introduced the fourth step catalyzed by double-bond reductase 2 (*DBR2*) (Zhang et al., 2008) and the fifth step, being the aldehyde dehydrogenase 1 (*ALDH1*) (Teoh et al., 2009). These five genes would yield dihydroartemisinic acid. The final conversion of dihydroartemisinic acid into artemisinin is thought to occur by photooxidation (Sy and Brown, 2002), thus the enzymatic end product is dihydroartemisinic acid. The five genes were transformed into *P. patens* using *in vivo* homologous recombination that allows multiple DNA fragments to be transformed at once into the genome (King et al., 2016). Here, we show that engineered *P. patens* can produce significant levels of artemisinin. This could become a sustainable and efficient production platform of artemisinin, which could potentially help to stabilize the supply of artemisinin and aid in containing malaria.

## Materials and Methods

### *P. patens* Material and Growth Conditions

*Physcomitrella patens* (Gransden ecotype, International Moss Stock Center #40001) was grown on solid and liquid PhyB media under sterile conditions, at 25°C with continuous 20–50 W/m^2^ light intensity. For PhyB media mix 800 mg Ca(NO_3_)_2_, 250 mg MgSO_4_⋅7H_2_O, 12.5 mg FeSO_4_⋅7H_2_0, 0.5 g (NH_4_)_2_C_4_H_4_O, 10 mL KH_2_PO_4_ buffer (25 g KH_2_PO_4_ per liter and adjusted to pH 6.5 with 4 M KOH), and 0.25 mL trace element solution (110 mg CuSO_4_⋅5H_2_0, 110 mg ZnSO_4_⋅7H_2_O, 1,228 mg H_3_BO_3_, 778 mg MnCl_2_⋅4H_2_O, 110 mg CoCl_2_⋅6H_2_O, 53 mg KI, 50 mg Na_2_MoO_4_⋅2H_2_O per liter). The medium can be solidified with 0.7% (w/v) agar and is sterilized by autoclaving at 121°C (Bach et al., 2014).

### Transformation of *P. patens*

A detailed description of *P. patens* transformation was previously published (Bach et al., 2014). Five days cultured *P. patens* with approximately 1.5 g (fresh weight) was digested by adding 1 mL of 0.5% DriselaseR enzyme solution in 8.5% mannitol (Sigma D9515) for every 40 mg of *P. patens* tissue. The tissue was incubated at room temperature with occasional gentle shaking for 30–60 min before filtering through a 100-µm pored mesh-filter. The filtrate was collected by centrifugation at 150–200 × *g* for 4 min with slow breaking. The pellet was washed twice with protoplast wash solution (8.5% mannitol, 10 mM CaCl_2_). Protoplast density was measured using a hemocytometer, and resuspended in MMM solution (9.1% d-mannitol, 10% MES, and 15 mM MgCl_2_) at a concentration of 1.6 × 10^6^ protoplasts/mL. 300 µL of the protoplast suspension and 300 µL of PEG solution were added to a 15-mL tube containing 10 µg total DNA followed by incubation in a water bath for 5 min at 45°C and another 5 min at room temperature. 300 µL of 8.5% d-mannitol were added five times followed by another five times dilutions with 1 mL of 8.5% d-mannitol. Transformed protoplasts were pelleted by centrifugation and resuspended in 500 µL of 8.5% d-mannitol and 2.5 mL of protoplast regeneration media (top layer; PRMT). 1 mL of the mixture was dispensed on three plates containing protoplast regeneration media (bottom layer; PRMB) overlaid with cellophane. The plates were incubated in continuous light for 5–7 days at 25°C. The cellophane and regenerating protoplasts were then transferred to PhyB media containing the appropriate selection marker for 2 weeks, before transferring on PhyB media without antibiotics for another 2 weeks. This process was repeated twice, after which the stable transformants was kept on PhyB media with biweekly subcultivation including blending in sterile water until further use.

### DNA Fragments and Genes

The Pp108 locus homologous recombination flanking regions were amplified from *P. patens* genomic DNA. The *ADS* gene was a kind gift from Assoc. Prof. Dae Kyun Ro, University of Calgary, Canada. The synthetic genes *CYP71AV1* (DQ268763), *ADH1* (JF910157.1), *DBR2* (EU704257.1), and *ALDH1* (FJ809784.1) were codon-optimized according to the *P. patens* codon usage by GenScript, USA. The Ubiquitin promoter and Ubiquitin terminator from *Arabidopsis thaliana* (CP002686.1) synthetic genes were also purchased from GenScript. The Maize Ubiquitin 1 promoter and G418 selection cassettes were obtained from the pMP1355 vector, a kind gift from Prof. Mark Estelle, University of California San Diego, USA. The rice actin promoter and hygromycin selection cassette were obtained from the pZAG1 vector, a kind gift from Assoc. Prof. Yuji Hiwatashi from Miyagi University, Japan.

### PCR, DNA Purification and Concentration

The DNA fragments were amplified using PhusionR High-Fidelity DNA Polymerase (New England Biolabs). The primers used are listed in Table S1 in Supplementary Material. PCR conditions and annealing temperatures were modified depending on primers and templates used in the reaction. PCR reactions using plasmid DNA as template were digested with DpnI (NEB, USA) for 1 h at 37°C followed by inactivation at 65°C for 20 min to lower background after transformation. PCR products were purified using QIAquick PCR Purification Kit (QIAGEN GmbH, Germany). The DNA fragments for transformations were concentrated *via* ethanol precipitation to a final concentration of ~1 μg/μL, determined using NanoDrop2000 (Thermo Fisher Scientific).

### GC–MS Analysis of Amorpha-4,11-Diene

Amorpha-4,11-diene production was measured using a Shimadzu GCMS-QP2010 Plus (GC-2010). The initial screening was performed by HS-SPME (Headspace-Solid Phase Micro-Extraction) (Drew et al., 2012; Andersen et al., 2015). Quantification of amorpha-4,11-diene was performed according to a published protocol (Rodriguez et al., 2014). One-week-old *P. patens* was blended in sterile water using a Polytron (PT 1200 E, Kinematic AG) to a final concentration of 0.2 g/mL (fresh weight). Two milliliters of the blended *P. patens* were inoculated into 20 mL liquid PhyB media and cultivated on a shaker under standard conditions for 4 days. Then, 2 mL of decane were added into the *P. patens* culture and cultivation was continued for up to 2 weeks. After 2 weeks, 100 µL of decane was harvested and diluted twice in ethyl acetate spiked with an internal standard (trans-caryophyllene), and analyzed by GC–MS. 1 µL of the extract was injected in split mode and separated with HP-5MS UI column (20.0 m × 0.18 mm × 0.18 µm) with hydrogen as a carrier gas. The GC program was as follows, injection temperature of 250°C, oven temperatures of 60°C for 3 min and 60–320°C at 40°C/min. The amorpha-4,11-diene concentration was calculated based on the calibration curve of the internal standard run in parallel (Rodriguez et al., 2014).

### *P. patens* Growth and Biomass Measurements

*Physcomitrella patens* lines were blended in sterile water and subcultivated onto PhyB. After 1 week, the *P. patens* was blended for 30 s in sterile water, normalized to a concentration of 0.2 g/mL (fresh weight) as previously described (Zhan et al., 2014; Pan et al., 2015). 2 mL of the blended *P. patens* was inoculated into 20 mL liquid PhyB media and cultivated on a horizontal shaker under standard conditions without aeration. The *P. patens* cultures were prepared in 3 replicates and harvested in 3, 6, 12, 15, and 18 days. The harvested *P. patens* tissue was dried overnight in an oven at 90°C and weighed.

### Metabolite Extraction and UPLC-MRM-MS Analysis

*Physcomitrella patens* lines were blended in sterile water and subcultivated onto PhyB. After 1 week the fresh *P. patens* samples were harvested, snap-frozen, and ground into a fine powder. Samples of 3,000 mg were extracted with 3 mL citrate phosphate buffer, pH 5.4, followed by vortexing and sonication for 15 min. 1 mL Viscozyme (Sigma V2010) was added and samples were incubated at 37°C. The whole mixture was then extracted three times with 3 mL ethyl acetate, concentrated to a volume of 1 mL, and stored at −20°C. For liquid culture extracts, 500 mL of liquid culture was harvested, passed through a filter paper and extracted with 200 mL of ethyl acetate in a separation funnel. Ethyl acetate was concentrated to a volume of 1 mL and stored at −20°C. Ethyl acetate of both liquid culture and *P. patens* sample extracts were then dried under a flow of N_2_ and resuspended into 300 µL of 75% MeOH:H_2_O (V:V). Extracts were passed through a 0.45-µm membrane filter (Minisart^®^ RC4, Sartorius, Germany) before analysis.

Artemisinin and artemisinin biosynthesis pathway intermediates were measured in a targeted approach by using a Waters Xevo tandem quadrupole mass spectrometer equipped with an electrospray ionization source and coupled to an Acuity UPLC system (Waters), essentially as described before (Ting et al., 2013). A BEH C18 column (100 mm × 2.1 mm × 1.7 µm; Waters) was used for chromatographic separation by applying a water:acetonitrile gradient. The gradient started with 5% (v/v) acetonitrile in water with formic acid [1:1,000 (v/v)] for 1.25 min, then raised to 50% in 2.35 min and further raised to 90% at 3.65 min. This was kept for 0.75 min before returning to the 5% acetonitrile/water (v/v) with formic acid [1:1,000 (v/v)] by using a 0.15-min gradient. The same solvent composition was used to equilibrate the column for 1.85 min. The flow rate was 0.5 mL/min, and the column temperature was maintained at 50°C. Injection volume was set to 10 µL. Desolvation and cone gas flow were set to 1,000 and 50 L/h, and the mass spectrometer was operated in positive ionization mode. Capillary voltage was set at 3.0 kV. Desolvation and source temperatures were set at 650 and 150°C, respectively. The cone voltage was optimized for all metabolites using the Waters IntelliStart MS Console. Fragmentation by collision-induced dissociation was done in the ScanWave collision cell using argon. Multiple Reaction Monitoring (MRM) was used for detection and quantification of artemisinin. MRM transitions for artemisinin and pathway intermediates measurement settings were optimized for MRM channels, which are presented in (Table S2 in Supplementary Material). Artemisinin and dihydroartemisinic acid were gifts from Dafra Pharma (Belgium). Other precursors were synthesized from dihydroartemisinic acid by Chiralix (Nijmegen, the Netherlands) and were then examined by NMR (>98% purity). External calibration curves were measured by using reference standards. The metabolite profiling was performed twice with 2 months apart, using the same original cell lines that had been subcultivated four times on PhyB media in between the analysis.

### Analysis of Conjugated Artemisinin Biosynthesis Pathway Intermediates by LC-QTOF-MS

In order to analyze the putative conjugated forms of artemisinin biosynthesis pathway intermediates, *P. patens* lines were blended in sterile water and subcultivated onto PhyB. After 1 week, 100 mg of fresh *P. patens* was ground in liquid nitrogen and extracted with 300 µL MeOH:formic acid [1,000:1 (v/v)]. Samples were briefly vortexed and sonicated for 15 min, followed by 15 min centrifugation at 13,000 × *g*. Extracts were passed through a 0.45-µm membrane filter (Minisart^®^ RC4, Sartorius, Germany) before analysis on a Water alliance 2795 HPLC connected to a QTOF Ultima V 4.00.00 mass spectrometer (Waters. MS technologies, UK). The mass spectrometer was operated in negative ionization mode. A precoloumn of 2.0 mm × 4 mm (Phenomenex, USA) was connected to the C18 analytical column (Luna 3 µm C18/2 100A; 2.0 mm × 150 mm; Phenomenex, USA). Degassed eluent A and B were HPLC-grade water:formic acid [1,000:1 (v/v)] and acetonitrile:formic acid [1,000:1 (v/v)], respectively. The flow rate was 19 mL/min. The HPLC gradient started from 5% eluent B and linearly increased to 75% in 45 min. after that the column was equilibrated for 15 min with 5% eluent B. 5 µL of each sample was used for injection.

### *P. patens* Cell Components Isolation and Extraction

*Physcomitrella patens* protoplasts were isolated according to a previously published protocol (Bach et al., 2014). The *P. patens* lines were blended in sterile water and subcultivated onto PhyB. After 1 week, protoplast were isolated and pelleted by centrifugation at 150–200 × *g* for 4 min with slow breaking. Supernatant, which contained the apoplast (AP) was harvested and 10 mL of ultra-pure water was added to the pelleted protoplast to lyse the cells. Lysed cells were centrifuged at 6,000 × *g* for 10 min, and 7 mL of the supernatant was mixed with an equal amount of sucrose to a final concentration of 0.3 M. The mixture was loaded into an ultracentrifuge tube with a 2 mL over-lay of water and centrifuged at 135,000 × *g* for 40 min at room temperature using a swinging bucket rotor. The bottom layers containing the cytoplasm (CT) and the cell pellet (CP) was harvested. The top layer (1.5 mL) containing neutral lipid bodies (LBs) was transferred to another centrifuge tube, mixed with sucrose to a final concentration of 0.3 M, overlaid with 2 mL of water as before, and 1 mL of the top-most layer of the LBs after centrifugation at 135,000 x *g* for 40 min. The harvested samples; AP, CT, LBs, and CP was then extracted with 3 mL ethyl acetate and concentrated to a volume of 1 mL and stored at −20°C. The samples were then dried under a flow of N_2_ and resuspended into 300 µL of 75% MeOH:H_2_O (V:V). Extracts were passed through a 0.45-µm membrane filter (Minisart^®^ RC4, Sartorius, Germany) before analysis.

## Results and Discussions

### Engineering of *P. patens*

We introduced *ADS* under the control of the strong constitutively expressed promoter, Ubiquitin1 from *Zea mays* with geneticin (G418) as resistance cassette. After two rounds of selection the *ADS* product amorpha-4,11-diene was detected in 11 lines with an average content of 200 mg/L, which is higher than in *A. annua* (Ma et al., 2009), *Nicotiana tabacum* (Wallaart et al., 2001), *E. coli* (Martin et al., 2003), and yeast (Ro et al., 2006). An encouraging initial result was achieved without further optimization of terpenoid biosynthesis. The best producing line was selected for further transformation with *CYP71AV1* and *ADH1*. The *CYP71AV1*-LP4/2A-*ADH1* construct was controlled by the rice actin promoter with hygromycin resistance cassette. The genome homologous overhang was targeted to remove the previously integrated G418 cassette during the recombination event. Of 47 transformed lines, 11 were chemo- and genotyped and one line was selected for transformation with the final genes, *DBR2* and *ALDH1*. The *DBR2*-LP4/2A-*ALDH1* construct was controlled by the Arabidopsis Ubiquitin promoter and the G418 selection cassette was used again. The hygromycin cassette was targeted for recombination to remove this selection marker. Three independent transformants were recovered, and genotyping showed that the five genes in the biosynthesis of dihydroartemisinic acid were integrated into the genome (Figure S1 in Supplementary Material). PCR analysis showed there was no untargeted integration for the selected lines. This showed that the selected *P. patens* lines have a uniform integration of the five genes and one copy of each.

### Metabolite Profiling and Growth of Engineered Strains

**Figure 2:**
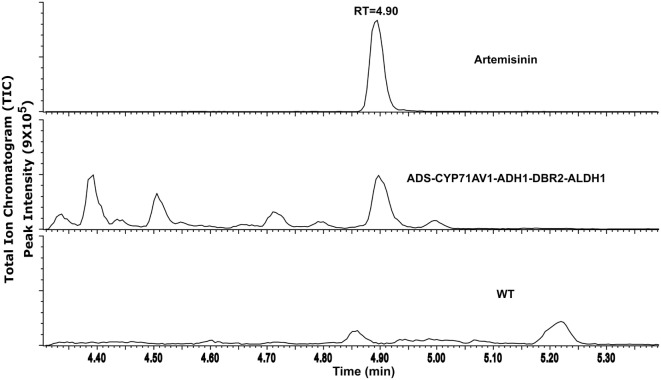
UPLC-MRM-MS analysis of artemisinin produced from transgenic *Physcomitrella patens*, an internal standard (artemisinin) and WT as control with retention time. TIC represents the sum of multiple reaction monitoring channels used for the detection of artemisinin: *m/z* 283.19 > 219.21; 283.19 > 247.19; and 283.19 > 265.22.

Extracts of the transgenic *P. patens* lines containing all five genes were analyzed using ultra-high performance liquid chromatography coupled with a triple quadrupole mass spectrometer operated in MRM mode (MRM) (UPLC-MRM-MS). MRM traces identical with the artemisinin standard were detected in the *P. patens* extracts, but not in the liquid culture medium or in extracts of wild-type *P. patens* (WT) (Figure 2; Figure S2 in Supplementary Material). The analysis was performed twice with 2 months apart in triplicates each time. The presence of artemisinin, but no intermediates, in *P. patens* was confirmed by comparison with an artemisinin standard (Figure S2 in Supplementary Material). Quantification using external calibration showed that the artemisinin yield was 0.21 mg/g dry weight (DW), which is in the range of native *A. annua* (0.001–1.54 mg/g DW) (Bhakuni et al., 2001) and in cross-bred plants (up to 10 mg/g DW) and higher in genetically engineered (up to 30 mg/g DW) (Tang et al., 2014). The amount of artemisinin in our *P. patens* lines is higher than what was initially obtained *via* heterologous expression in *N. tabacum* (0.0068 mg/g DW) (Farhi et al., 2011) and in *Nicotiana benthamiana* (0.003 mg/g DW) (Wang et al., 2016). Recent efforts of targeting the biosynthesis to the chloroplast elevated the yield in *N. tabacum* to 0.8 mg/g DW (Malhotra et al., 2016), since glycosylation of the precursors was avoided. Glycosylation and glutathione conjugation was previously shown to influence the yield in *N. benthamiana* (Mukanganyama et al., 2001; van Herpen et al., 2010; Ting et al., 2013; Liu et al., 2014). We also explored the presence of sugar and glutathione conjugated products by liquid chromatography coupled to quadrupole time-of-flight mass spectrometry (LC-QTOF-MS). However, no glycosylated or glutathione conjugated products were detected in the culture medium nor in the *P. patens* extracts. The lack of glycosides may be explained by the fact that *P. patens* only has 20 glycosyltransferase 1 (*GT1*) genes that encode for enzymes involved in deactivation or detoxification of secondary metabolites, while higher vascular plants usually have hundreds (Yonekura-Sakakibara and Hanada, 2011). As no pathway intermediates (conjugated or not) were detected in our extracts, we conclude that the pathway operates efficiently in *P. patens*.

**Figure 3:**
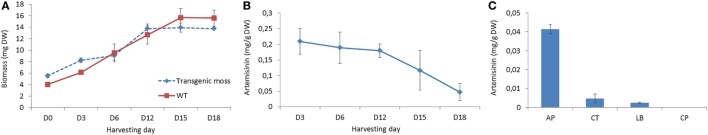
Growth and production of artemisinin in *Physcomitrella patens*. **(A)** Time course analysis of growth of transgenic and WT *P. patens* in liquid culture. D0 indicates the initial sub-culturing day. **(B)** Time course analysis of artemisinin production in *P. patens*. Error bars denote the SE of triplicate shake-flask liquid culture. **(C)** Production of artemisinin after 18 days of cultivation in different cellular component of AP, apoplast; CT, cytoplasm; LBs, lipid bodies; and CP, cell pellet.

A slight reduction in growth rate was observed after day 12, resulting in 11% lower biomass in the transgenic line after 18 days (Figure 3A). This indicates that there is just a small disruption of other metabolic pathways responsible for *P. patens* growth and fitness. The effect of artemisinin biosynthesis on *P. patens* growth is less, but follows and suggests the same pattern as was previously observed (Simonsen et al., 2009; Zhan et al., 2014; Pan et al., 2015). The lesser growth is likely due to toxicity of the product and to depletion of nutrients in the media, but this requires further studies in semi-continuous cultures. The highest concentration (0.21 mg/g DW) of artemisinin was observed after 3 days of cultivation (Figure 3B), whereas the highest accumulative amount of artemisinin (2.5 mg artemisinin) was observed at day 12. This is mainly due to the increase in biomass. This show that the primary production of artemisinin in *P. patens* happens within the first 2 weeks after subcultivation. This rapid production of artemisinin is very valuable for future industrial production and suggests that a semi-continuous batch cultivation with weekly extraction and disruption of the cells can provide high amounts of artemisinin.

Conversion of dihydroartemisinic acid to artemisinin in *A. annua* has been suggested to occur *via* photooxidation and induced by oxygen (Sy and Brown, 2002). In the present study, *P. patens* was grown under 24-h light and ambient air, which seems to facilitate the conversion to artemisinin as dihydroartemisinic acid was not detected in the extract. The reduction in artemisinin content from day 12 to day 18 might be due to chemical degradation of the compound, but no obvious breakdown products was found in the analysis; thus, this drop requires further studies.

### Storage of Artemisinin in *P. patens*

Artemisinin biosynthesis occurs in the glandular trichomes of *A. annua*, but *P. patens* does not have trichomes. To identify the storage location of artemisinin in our transgenic *P. patens*, extracts of the AP, CT, LBs, and CPs were analyzed by UPLC-MRM-MS. Artemisinin was detected in all the extracts except for the CPs (Figure 3C). The highest accumulation was in the AP at 0.04 mg/g DW (after 18 days of cultivation, analyzed at 18 days to obtain enough biomass for the analysis) with 10- and 20-fold less in the CT and LBs. In the native plant *A. annua*, dihydroartemisinic acid is transported to the subcuticular space of the glandular trichomes before photooxidation into artemisinin (Brown, 2010). The high accumulation of artemisinin in the *P. patens* AP indicates a transport of pathway products over the cell membrane, as also shown *N. benthamiana* (Wang et al., 2016).

This work demonstrates a stable production of artemisinin in a photosynthetic organism that allows for large-scale industrial production. The production will not be affected by environmental and ecological variables and overall have a lower environmental impact than field production (water usages, fertilizers, petrol usages, etc.). It should be noted that besides using codon-optimized sequences, no other enhancement, e.g. increasing terpenoid precursor supply or multi copy gene integration was applied. The high flux through the terpenoid pathway is likely to be due to the metabolic robustness of *P. patens* (Schaefer and Zrÿd, 1997). In contrast, yeast needed extensive and complex precursor pathway engineering prior to the introduction of the artemisinic acid pathway genes. Further optimization of the metabolic network in *P. patens* has been shown to optimize sesquiterpenoid production (Zhan et al., 2014) and possibly also artemisinin production. Possible targets include overexpression of the key enzymes, 3-hydroxy-3-methylglutaryl-CoA reductase (*HMGR*), and farnesyl diphosphate synthase (*FPS*), which improved terpenoid production in other plants (van Herpen et al., 2010) and microbial (Ro et al., 2006; Paddon et al., 2013) hosts. Another possibility is targeting biosynthesis to different cellular compartments, which has also been shown to improve artemisinin and other sesquiterpenoid yield up to 1,000-fold in *N. tabacum* (Wu et al., 2006; Malhotra et al., 2016), which alone in *P. patens* would lead to a yield of 210 mg/g DW.

Optimization of artemisinin yield in *P. patens* can potentially result in a stable, sustainable, environmentally friendly, and commercially viable production platform. A considerable advantage of *P. patens* as an artemisinin production platform is that the extract only requires simple purification steps (Figure 2). This is different from the current yeast production platform that requires further chemical synthesis to yield artemisinin (Paddon et al., 2013; Turconi et al., 2014) and the production in *N. tabacum* was only described as semi-pure extracts (Malhotra et al., 2016). The use of *P. patens* could lead to a reduced price for artemisinin-based treatments, allowing lower income communities most affected by malaria, to contain malaria. Furthermore, scaling up the production of artemisinin in plant-based bioreactors would expand the use of plant cells in bioreactors.

## Conclusion

All five artemisinin biosynthetic pathway genes were engineered into the moss *P. patens*. *In vivo* biosynthesis of artemisinin was obtained without further modifications and a high initial production of 0.21 mg/g DW artemisinin was observed after only 3 days of cultivation. This bioengineering achievement expands the frontiers of synthetic biotechnology, offering a genetically robust plant-based platform, which can be scaled up for industrial production of other complex high-value plant-based compounds. *P. patens* uses light as an energy source, thus is potentially more cost effective than other carbon supplemented biotechnological platforms.

## Author Contributions

NI planned and performed the experiments, analyzed the data, and wrote the manuscript. ABK performed the UPLC-MRM-MS and LC-QTOF-MS, analyzed the data, and reviewed the manuscript. AP performed the *P. patens* cell components isolation experiments and reviewed the manuscript. ARvdK and HB reviewed the manuscript. HS planned the experiment and supervised and reviewed the manuscript.

## Conflict of Interest Statement

All authors declare that they have no conflict of interest. HS is co-founder of Mosspiration Biotech IVS that aim to produce fragrances in *P. patens*, but not artemisinin.
