# Supplementary material for: Stable Production of the Antimalarial Drug Artemisinin in the Moss Physcomitrella patens
Source: Front Bioeng Biotechnol. 2017 Aug 15;5:47. doi: 10.3389/fbioe.2017.00047 (PMC5559433; doi:10.3389/fbioe.2017.00047)
Supplement: Supplementary file 1 [file Data_Sheet_1.PDF]

Supplemental data

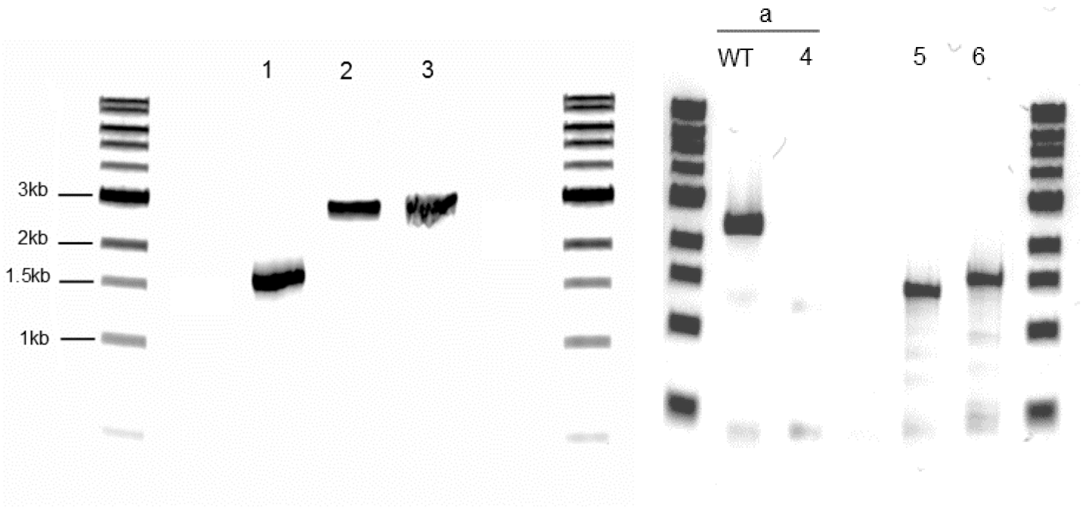

Supplemental figure 1. Transgenic moss line expressing the five genes via PCR using gene-specific primers of 1. *ADS* (1641bp), 2. *CYP71AV1*-LP4/2A-*ADH1* (2709bp) and 3. *DBR2*-LP4/2A-*ALDH1* (2748bp). Primer pair 'a' only bind at the DNA region specific to the wild type (WT) Pp108 locus, giving a band of 2580 bp if the WT locus is intact. This band is absent in the shown transformed line (4). Primer pair 5 and 6 binds at the recombinant region upstream and downstream the 5' and 3' Pp108 genomic locus giving sizes of 1442 and 1525 bp respectively, indicating insertion in the targeted genome locus. The PCR products were sequence-verified.

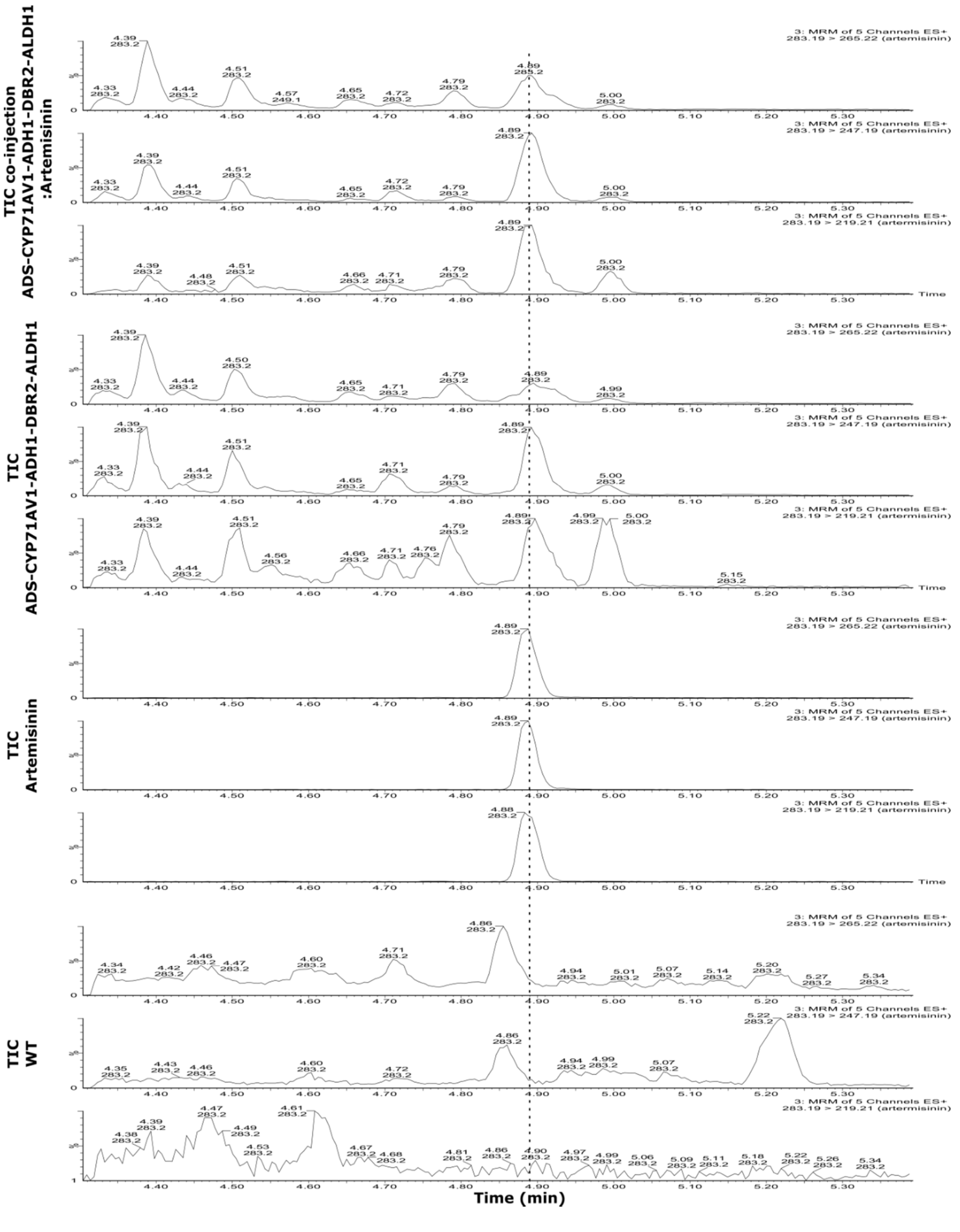

Supplemental figure 2. UPLC-MRM-MS analysis of artemisinin detection in three transition states (from bottom: WT *P. patens*, transgenic *P.patens*, artemisinin standard and co-injection of transgenic *P. patens*: artemisinin standard (1:1)).

Supplemental Table 1. Primers used

	Primer Sequence	Comment
1	TCAGAATTAGATTGACATATATGTTGAAAATGGATCAAAG	Forward primer for HR 5’ flanking region
2	ATTCCATTCTTGGTCAGATGAGTTTACTCTTTC	Reverse primer for HR 5’ flanking region
3	TAATTCTTTCTTTTTGAGGTATATATTATCTTAGCATGG	Forward primer for HR 3’ flanking region
4	ACGAAGGCCGTTCTTCCCTGG	Reverse primer for HR 3’ flanking region
5	CCCTGTTGTTTGGTGTTACTTCTGCAGGTCGAAGCTAAATGGGCTAACGAAGGC	Forward primer for ADS
6	GGCGTCTCGCATATCTCATTAAAGCAGGACTCAGATGGACATCGGGTAAACCAG	Reverse primer for ADS
7	TAATGAGCATTGCATGTCTAAGTTATAAAAAATTACCAC	Forward primer for ZmUBI promoter
8	AAATAATTATAAAACATACTTGTTTATTATAATAGATAGGTACTCAAGGTTAGAGC	Reverse primer for ZmUBI promoter
9	GTCTCGCATATCTCATTAAAGCAGGAC	Forward primer for OCS terminator
10	GTTACCCGGCCGCCGTCCTCAAAAAGAAAGAATTA	Reverse primer for OCS terminator
11	CTACTCCAAAAATGTCAAAGATACAGTCTCAGAAG	Forward primer for G418 selection casette
12	ACTGGATTTTGGTTTTAGGAATTAGAAATTTTATTGATAGAAG	Reverse primer for G418 selection casette
13	GGCCCGAGGTCATTCATATGC	Forward primer for rice actin promoter
14	GTGCCATTGCTTTGAGGATAGATTTCATTCTAGAGGATCCCCGATATCTTCTACC	Reverse primer for rice actin promoter
15	ATGAAATCTATCCTCAAAGCAATGGCAC	Forward primer for CYP71ADH1-LP4/2A-ADH1
16	AGTAGCAACTTCGTCTGCTGCATTTGATCAAAACTTAATAAGGATTTTCACGCAGTCAGG	Reverse primer for CYP71ADH1-LP4/2A-ADH1
17	AGCGGCCGATCGTTCAAAC	Forward primer for NOS terminator
18	GAGACTGTATCTTTGACATTTTTGGAGTATTAGCATTCTTTCTGAAAGGGAATTCTCATG	Reverse primer for NOS terminator
19	TACTCCAAAAATGTCAAAGATACAGTCTCAGAAG	Forward primer for Hyg selection casette
20	AGTTTTGATCTTGAAAGATCTTTTATCTTTAGAGTTAAGAACTCTT	Reverse primer for Hyg selection casette
21	ACAACCAAGCGGCTTGAAACAATAG	Forward primer for AtiUBI promoter
22	GGAGAACAGAGTAGGTTTTTCGGACATCTTTTGTGTTTCGTCTTCTCTCACGTAGAAAC	Reverse primer for AtiUBI promoter
23	ATGTCCGAAAAACCTACTCTGTTCTCC	Forward primer for DBR2-LP4/2A-ALDH1
24	AGCAAGCAAGAGATGGGATTCTTGATAAGAGTCTCTTCACAGCCACGGACTGTCATAG	Reverse primer for DBR2-LP4/2A-ALDH1
25	AGAGACTCTTATCAAGAATCCCATCTCTTGC	Forward primer for Arabidopsis terminator
26	ACTGTATCTTTGACATTTTTGGAGTAGAGTTTGGTACGTCACAAACTTAAATCATTTTAC	Reverse primer for Arabidopsis terminator
27	CCAATCCGACCTCAAACAGAGC	Forward primer for 5’ Pp108 locus
28	TGCTGCATTATGGATGGAAG	Reverse primer for 3’ Pp108 locus
29	TGTGTTGAGCATATAAGAAACCCTTAGTATGTA	Forward primer at Tcamv terminator
30	CAGTGACGACAAATCGTTGG	Reverse primer between OCS terminator and 3’ 108 locus downstream

Supplemental Table 2. Optimized MRM transition settings for UPLC-MRM-MS measurement of artemisinin, artemisinic acid, dihydroartemisinic acid, artemisinic alcohol and dihydroartemisinic alcohol.

	Parent (*m/z*)	Daughter (*m/z*)	Cone voltage	Collision voltage
Artemisinin	283.19	265.22	12	14
		247.19	12	8
		219.21	12	8
Artemisinic acid	235.16	217.21	18	14
		199.25	18	16
		189.22	18	10
Dihydroartemisinic acid	237.16	163.17	16	28
		107.12	16	26
		81.10	16	18
Artemisinic alcohol	221.16	203.27	14	20
		147.09	14	10
Dihydroartemisinic alcohol	223.22	205.27	14	24
		109.13	14	14
		95.07	14	12
